# Current knowledge on bioacoustics of the subfamily Lophyohylinae (Hylidae, Anura) and description of Ocellated treefrog *Itapotihyla langsdorffii* vocalizations

**DOI:** 10.7717/peerj.4813

**Published:** 2018-05-31

**Authors:** Lucas Rodriguez Forti, Roseli Maria Foratto, Rafael Márquez, Vânia Rosa Pereira, Luís Felipe Toledo

**Affiliations:** 1 Laboratório Multiusuário de Bioacústica (LMBio) e Laboratório de História Natural de Anfíbios Brasileiros (LaHNAB), Departamento de Biologia Animal, Instituto de Biologia, Universidade Estadual de Campinas, Campinas, São Paulo, Brazil; 2 Fonoteca Zoológica, Departamento de Biodiversidad y Biología Evolutiva, Museo Nacional de Ciencias Naturales, CSIC, Madrid, Spain; 3 Centro de Pesquisas Meteorológicas e Climáticas Aplicadas à Agricultura (CEPAGRI), Universidade Estadual de Campinas, Campinas, SP, Brazil

**Keywords:** Anura, Bioacoustics, Call repertoire, Hylidae, Taxonomy, Vocalization

## Abstract

**Background:**

Anuran vocalizations, such as advertisement and release calls, are informative for taxonomy because species recognition can be based on those signals. Thus, a proper acoustic description of the calls may support taxonomic decisions and may contribute to knowledge about amphibian phylogeny.

**Methods:**

Here we present a perspective on advertisement call descriptions of the frog subfamily Lophyohylinae, through a literature review and a spatial analysis presenting bioacoustic coldspots (sites with high diversity of species lacking advertisement call descriptions) for this taxonomic group. Additionally, we describe the advertisement and release calls of the still poorly known treefrog, *Itapotihyla langsdorffii*. We analyzed recordings of six males using the software Raven Pro 1.4 and calculated the coefficient of variation for classifying static and dynamic acoustic properties.

**Results and Discussion:**

We found that more than half of the species within the subfamily do not have their vocalizations described yet. Most of these species are distributed in the western and northern Amazon, where recording sampling effort should be strengthened in order to fill these gaps. The advertisement call of *I. langsdorffii* is composed of 3–18 short unpulsed notes (mean of 13 ms long), presents harmonic structure, and has a peak dominant frequency of about 1.4 kHz. This call usually presents amplitude modulation, with decreasing intensity along the sequence of notes. The release call is a simple unpulsed note with an average duration of 9 ms, and peak dominant frequency around 1.8 kHz. Temporal properties presented higher variations than spectral properties at both intra- and inter-individual levels. However, only peak dominant frequency was static at intra-individual level. High variability in temporal properties and lower variations related to spectral ones is usual for anurans; The first set of variables is determined by social environment or temperature, while the second is usually related to species-recognition process. Here we review and expand the acoustic knowledge of the subfamily Lophyohylinae, highlighting areas and species for future research.

## Introduction

The acoustic repertoire of a species may include signals encoding information about physiological state, location and social status (Snowdon, 2011). In anurans, the most prevalent acoustic signal is the advertisement call, which is used, at least, for mate attraction, sexual selection and territorial defense (Duellman, 1970; Wells, 2007; Wells & Schwartz, 2007). As a crucial element of reproductive isolation (Gerhardt, 1992; Ryan & Rand, 2001; Padial et al., 2008), such acoustic signals present properties related to species recognition (Ryan & Rand, 2001; Wells & Schwartz, 2007). In this context, the divergent evolution between sister species may modulate acoustic differences (Wilkins, Seddon & Safran, 2013). Generally, spectral properties, such as dominant and minimum frequency, are body size dependent (Gingras et al., 2013) and related to the process of conspecific recognition, since these variables tend to be static (i.e., presenting lower intraspecific variation) (*sensu* Gerhardt, 1991). However, temporal properties, such as call duration and pulse rate, in turn, show higher variability (Márquez & Eekhout, 2006; Briggs, 2010; Forti, Strüssman & Mott, 2010; Kaefer & Lima, 2012; Forti, Márquez & Bertoluci, 2015; Forti et al., 2016), and are determined by social and climatic conditions (Wong et al., 2004; Lingnau & Bastos, 2007).

Another important acoustic signal for the mate recognition system is the release call, which acts as a negative response to male mating attempt (Toledo et al., 2015). This signal may provide information about the identity of the sender (Sullivan, 1989; Duellman & Trueb, 1994; Castellano et al., 2002), which justifies their formal descriptions as being potentially useful for species diagnosis, taxonomic and phylogenetic studies (Brown & Littlejohn, 1972; Di Tada, Martino & Sinsch, 2001; Köhler et al., 2017).

In order to better understand the taxonomy and the diversity of anuran groups of interest, review articles including comparisons of vocalization properties are important (e.g., Hepp, Lourenço & Pombal Jr, 2017; Forti, Martins & Bertoluci, 2012; Forti, Márquez & Bertoluci, 2015; Forti, Lingnau & Bertoluci, 2017). In addition, the identification of geographical gaps is of great value for future research. Here we apply such an approach for a subfamily of hylid frogs (Lophyohylinae), in which only a fraction of species have their calls formally described. This subfamily has 85 species within 12 genera, which are widely distributed in South and Central America (Frost, 2018).

As in many other Neotropical species, the acoustic repertoire of *Itapotihyla langsdorffii* (Dumeril & Bibron, 1841), a species in the subfamily Lophyohylinae, is poorly studied and only a short description of the advertisement call from a population from Argentina (far from its type locality) was published (Straneck, Olmedo & De Carrizo, 1993). This is a monotypic taxon described from “baixada fluminense”, municipality of Rio de Janeiro (Frost, 2018). However, the species has a wide distribution with populations occurring along the Atlantic forest (and peripheral areas in the Cerrado Biome), in Brazil from the state of Rio Grande do Sul to the state of Sergipe (Cazelli & Moura, 2012). Populations also occur in northeastern Argentina and southeastern Paraguay (Frost, 2018). Most recent phylogenies place *Itapotihyla langsdorffii* as sister to species in the genera *Aparasphenodon, Argenteohyla, Corythomantis, Dryaderces, Nyctimantis, Osteocephalus, Osteopilus, Phyllodytes, Phytotriades, Tepuihyla* and *Trachycephalus* (Duellman, Marion & Hedges, 2016; Frost, 2018).

Herein we present an overview on the acoustic knowledge of the subfamily Lophyohylinae, pointing to new directions for recording efforts, and describe the advertisement and release calls of *Itapotihyla langsdorffii* from southeastern Brazil, reporting variations of its call properties.

## Methods

### Identification of *bioacoustic coldspots* and acoustic data of Lophyohylinae

**Figure 1:**
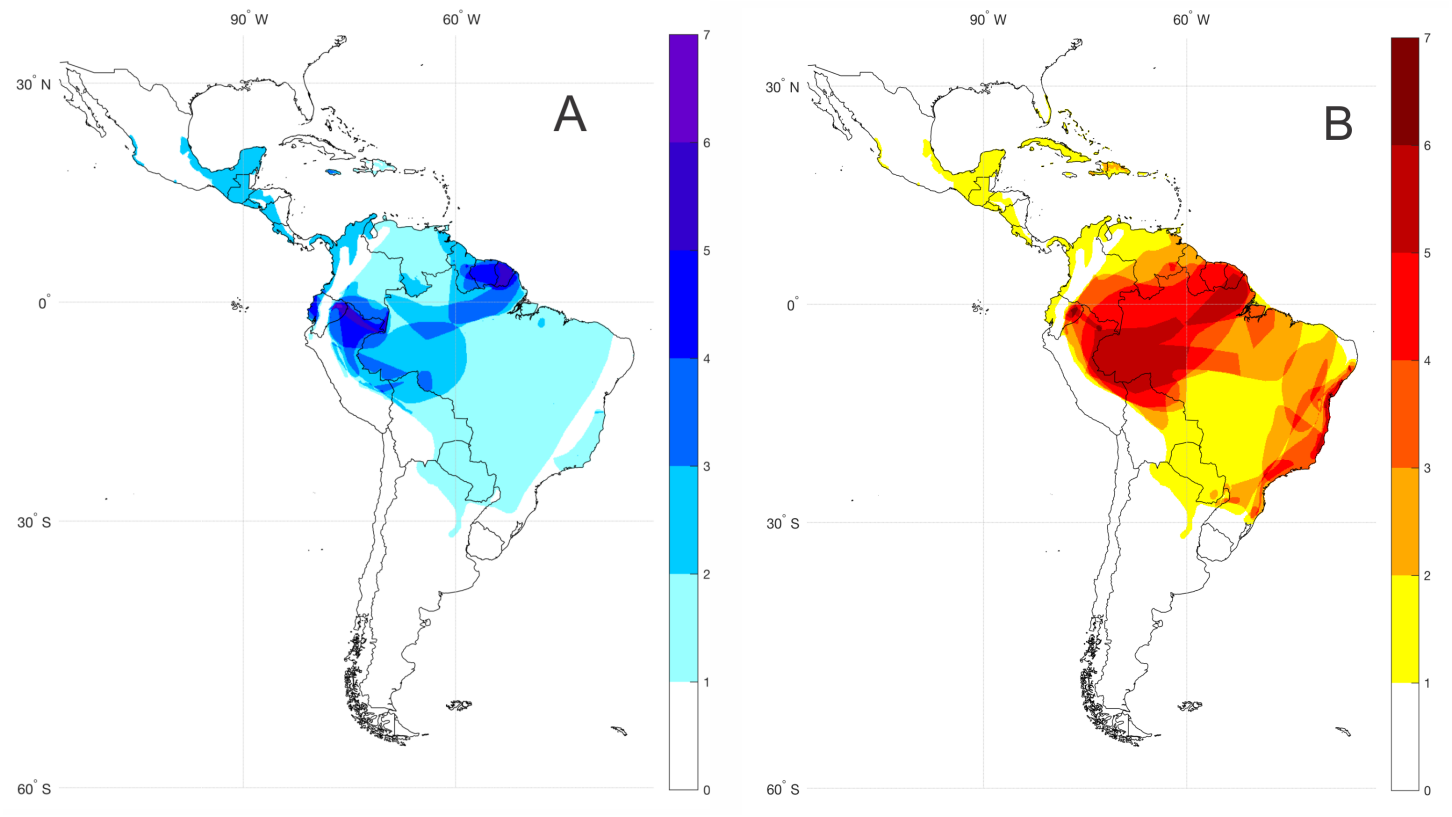
Bioacoustic coldspots and species richness hotspots for the subfamily Lophyohylinae. (A) Bioacoustic coldspots and (B) species richness hotspots for the subfamily Lophyohylinae. Darker colors represents greater density of species.

For the spatial analysis of *bioacoustic coldspots* (=areas with the highest number of species lacking call descriptions) of the subfamily Lophyohylinae, we compiled the geographical distribution dataset of species with unknown vocalizations based on the IUCN (2017) database and articles with available information on the species geographical distribution. We created shape files editing minimum convex polygons of each species distribution using the software ArcMap 10.5. Each species occurrence shape file was interpolated in a grid (0.25 degree resolution) Boolean matrix spanning the latitude and longitude range 36°N–60°S; 116°W–132°W. The Boolean matrixes for all species are summed and result in the agglomeration species map, as shown in Fig. 1.

We reviewed data from the available literature (scientific journal articles and text books using web search tools, as Google search, Google Scholar and ResearchGate, see Table 1) that present call descriptions of the species within the subfamily Lophyohylinae. This allowed us to compile the information into a comparative table with acoustic data for 39 species.

**Table 1:** Advertisement call properties of species in the Lophyohylinae subfamily. Values are present as mean ± SD (range).

**Species**	**Simple/ complex call**	**Note type**	**Tonal/ Pulsed**	**Notes per call**	**Duration call (s)**	**Internote interval (s)**	**Note duration (s)**	**Mininum frequency (Hz)**	**Maximum frequency (Hz)**	**Peak dominant frequency (Hz)**	**Reference**
*Aparasphenodon arapapa*	Simple	–	Pulsed	–	–	–	0.15 ± 0.02 (0.13–0.18)	604.82 ± 24.56 (557.9–647.2)	2,122.42 ± 212.9 (1,847.7–2,588.9)	1,481.52 ± 36.30 (1,464.3–1,550.4)	Lourenço-De-Moraes et al. (2013)
*Argenteohyla siemersi*	Simple	–	Pulsed	–	1.180–5.420	–	–	–	–	(1,700–2,070)	Cajade et al. (2010)
*Corythomantis greeningi*	Simple	A	Pulsed		0.252 ± 0.047 (0.079–0.3420)		0.252 ± 0.047 (0.079–0.3420)			860 ± 98 (730–1,250)	Juncá, Carneiro & Rodrigues (2008)
*Corythomantis greeningi*	Simple	B	Pulsed				0.071 ± 0.013 (0.039–0.1)			980 ± 210 (730–1,460)	Juncá, Carneiro & Rodrigues (2008)
Itapotihyla langsdorffii^*^	Simple	–	Tonal	11.7 ± 7.4 (3–36)	0.675 ± 0.211 (0.763–2.796)	0.0713 ± 0.06 (0.006–0.61)	0.035 ± 0.016 (0.009–0.103)	1,086 ± 201 (187–1,507)	2,499 ± 279 (1,406–3,531)	(1,033–2,799)	This study
*Nyctimantis rugiceps*	Simple	–	Pulsed	–	–	–	–	–	–	847 (478–1,130)	Duellman & Trueb (1976)
*Osteocephalus buckleyi*	Simple	–	Pulsed	–	–	–	–	–	–	745.66 ± 0.87 (745.04–746.28)	Ron et al. (2012)
*Osteocephalus cannatellai*	Simple	–	Tonal	–	–	–	–	–	–	1,049.54 ± 247.18 (771.6–1,412.6)	Ron et al. (2012)
*Osteocephalus deridens*	Simple	–	Pulsed	12.8 (9–16)	2.40 (1.61–3.25)	0.142 (0.077–0.22)	0.061 (0.037–0.115)	–	–	(2,340–2,440)	Jungfer et al. (2000)
*Osteocephalus leprieurii*	Simple	–	Pulsed	7	–	–	0.2352 (0.2189–0.2568)	–	–	2,083 (1,635.6–2,342.3)	De La Riva, Márquez & Bosch (1995)
*Osteocephalus leprieurii*	Complex	Type 1	Pulsed and Tonal	–	–	–	0.1143 (0.099–0.127)	–	–	(1,036–1,740)	Jungfer & Hödl (2002)
*Osteocephalus leprieurii*	Complex	Type 2	Pulsed and Tonal	–	–	–	0.0675 (0.0618–0.0779)	–	–	(1,500–2,900)	Jungfer & Hödl (2002)
*Osteocephalus mutabor*	Simple	–	Pulsed	12.5 (9–19)	4.41 (3.02–6.43)	0.17 (0.11–0.30)	–	–	–	(860–1,300)	Jungfer & Hödl (2002)
*Osteocephalus taurinus*	Simple	–	Pulsed	8	–	–	0.7615 (0.7116–0.8461)	–	–	1,501.2 (1,446.2–1,551)	De La Riva, Márquez & Bosch (1995)
*Osteopilus dominicensis*	Simple	–	Pulsed	1	0.28 ± 0.08 (0.06–0.63)	77 ± 0.001 (15–150)	–	–	–	2,058 ± 233 (1,500–2,620)	Galvis et al. (2016)
*Osteopilus marinae*	Simple	–	Pulsed	(15–17)	1.70	–	–	–	–	2,400	Hedges (1987)
*Osteopilus pulchrilineatus*	Complex	A	Pulsed	(1–2)	10.69 ± 6.7 (3.8–31)	0.89 ± 0.41 (0.054–3.5)	–	–	–	2,950 ± 600	Galvis et al. (2016)
*Osteopilus pulchrilineatus*	Complex	B	Pulsed	(2–20)	–	–	–	–	–	2,060 ± 430	Galvis et al. (2016)
*Osteopilus septentrionalis*	Complex	A	Pulsed	–	–	–	0.15	–	–	2,300	Blair (1958)
*Osteopilus septentrionalis*	Complex	B	Pulsed	–	–	–	0.35	–	–	–	Blair (1958)
*Phyllodytes amadoi*	Simple	–	Pulsed	13–17	3.41 ± 0.28 (2.99–4.10)	0.204 ± 0.02 (0.137–0.285)	0.043 ± 0.021 (0.008–0.119)	–	–	3,962 ± 192.6 (3,789.8–4,306.6)	Vörös, Dias & Solé (2017)
*Phyllodytes acuminatus*	Simple	–	Tonal	1–4	0.10 ± 0.03 (0.03–0.17)	–	–	–	–	2,070 ± 4,570	Campos et al. (2014)
*Phyllodytes edelmoi*	Simple	–	Pulsed	26.46 ± 2.33 (22–29)	5.2 ± 0.44 (4.28–5.73)	–	0.1 ± 0.003 (0.044–0.163)	–	–	2,840 ± 160 (1,490–3,320)	Lima, Lingnau & Skuk (2008)
*Phyllodytes gyrinaethes*	Simple	–	Pulsed	4.90 ± 0.60 (4–6)	1.70 ± 0.30 (1.30–2.30)	–	0.04 ± 0.01 (0.02–0.07)	–	–	2,750 ± 1,600 (2,530–3,090)	Roberto & Ávila (2013)
*Phyllodytes kautskyi*	Simple	–	Tonal	21	3.55 ± 0.19	(0.06–0.12)	0.085 ± 0.012	–	–	1,370 (870–1,810)	Simon & Gasparini (2003)
*Phyllodytes kautskyi*	Simple		Tonal	(21–22)	(3.48–3.90)	(0.08–0.14)	0.074 ± 0.014			1,160 (880–1,620)	Simon & Peres (2012)
*Phyllodytes luteolus*	Simple	–	Pulsed	8–15	5.0	–	0.125	–	–	(2,000–6,000)	Weygoldt (1981)
*Phyllodytes megatympanum*	Simple		Tonal	13.37 ± 2.56 (10–19)	5.91 ± 4.56 (3.20–23.63)	0.305 ± 0.10 (0.10–0.61)	0.092 ± 0.08 (0.009–0.245)			3,980 ± 136 (3,560–4,120)	Marciano-Jr, Lantyer-Silva & Solé (2017)
*Phyllodytes melanomystax*	Simple	–	Tonal	1	0.07 ± 0.04	–	0.07 ± 0.04	–	–	(1,390–3,360)	Nunes, Santiago & Juncá (2007)
*Phyllodytes praeceptor*	Simple	–	Pulsed	8.39 ± 1.55 (6–12)	5.34 ± 1.53 (3.02–9.41)	–	–	–	–	3,045 ± 115 (2,928–3,273)	Orrico, Dias & Marciano-Jr (2018)
*Phyllodytes tuberculosus*	Simple	–	Pulsed	18.60 ± 3.36 (14–23)	6.72 ± 1.73 (4.65–9.35)	0.21 ± 0.048 (0.07–0.036)	0.17 ± 0.047 (0.07–0.25)	–	–	2,460 ± 4,500 (1,680–3,270)	Juncá et al. (2012)
*Phyllodytes wuchereri*	Simple	–	Pulsed and Tonal	16.18 ± 3.25 (10–21)	4.7 ± 1.23 (2.75–6.75)	0.12 ± 0.02 (0.09–0.21)	0.19 ± 0.04 (0.11–0.32)	–	–	1,350 ± 100 (1,290–1,460)	Cruz, Marciano-Jr & Napoli (2014)
*Phyllodytes wuchereri*	Simple		Pulsed	18 ± 2 (16–20)	4.30 ± 0.30 (3.90–4.70)	0.12 ± 0.019 (0.087–0.195)	0.12 ± 0.013 (0.049–0.140)			3,250 ± 80 (3,190–3,450)	Magalhães, Juncá & Garda (2015)
*Tepuihyla edelcae*	Simple	–	Tonal	(1–4)	0.25 (0.03–0.4)	0.025	0.03	–	–	1,458.8 (1,382.8–1,523.4)	Myers & Donnelly (2008)
*Tepuihyla obscura*	Simple	–	Tonal	2 (1–3)	0.18 (0.02–0.83)	0.09 (0.01–0.13)	0.08 (0.04–0.11)	–	–	1,207.29 (775.2–1,378.1)	Kok et al. (2015)
*Tepuihyla rodriguezi*	Simple	–	Tonal	(8–21)	(0.69–1.64)	–	0.041 (0.013–0.42)	–	–	1,489.2 (624.5–2,624.1)	Kok et al. (2015)
*Tepuihyla shushupe*	Simple		Tonal	56–59	16.7 ± 0.47 (16.4–17.2)					515.6	Ron et al. (2016)
*Tepuihyla tuberculosa*	Simple		Tonal	35	(12.2–13.1)					(562.5–632.8)	Ron et al. (2016)
*Trachycephalus atlas*	Simple	–	Pulsed	12.4 ± 1.0 (10–15)	0.17 ± 0.02 (0.14–0.22)	0.009 ± 0.003 (0.007–0.015)	0.005 ± 0.002 (0.003–0.006)	–	–	1,840 ± 700 (1,690–1,880)	Santos-Silva, Ferrari & Juncá (2012)
*Trachycephalus cunauaru*	Simple	–	Pulsed and Tonal	2.08 ± 0.17 (1–3)	–	0.47 ± 0.17 (0.31–0.96)	0.47 ± 0.06 (0.26–0.55)	300 ± 50 (210–380)	1,980 ± 490 (1,520–2,690)	830 ± 360 (390–1,400)	Gordo et al. (2013)
*Trachycephalus dibernardoi*	Simple	–	Pulsed	–	(0.35–0.55)	(0.8–3.0)	(0.470–0.760)	–	–	1,550 (1,100–1,800)	Kwet & Solé (2008)
*Trachycephalus imitatrix*	Simple	–	Pulsed	1	0.1 ± 0.02	–	–	477.9 ± 58.1	1,859.7 ± 126	999.1 ± 206.2	Garey (2012)
*Trachycephalus nigromaculatus*	Simple	–	Pulsed	1	0.16 ± 0.007 (0.15–0.17)	–	–	–	–	(1,290–1,990)	Abrunhosa, Wogel & Pombal Jr (2001)
*Trachycephalus resinifictrix*	Simple	–	Tonal	3.3 (1–6)	–	0.633 (0.471–0.809)	0.307 ± 26.15 (0.249–0.366)	–	1,300	–	Hödl (1991)
*Trachycephalus typhonius*	Simple	–	Tonal	–	–	–	(0.350–0.550)	2,325	2,842	(1,800–2,500)	Zimmerman & Hödl (1983)

**Notes.**

* Indicates that the average number for this acoustic variable has no meaning, since peak dominant frequency can be in the first or second harmonic and the average number gives us a frequency position between the harmonics.

### Acoustic analysis

Call recordings of *Itapotihyla langsdorffii* were made on 31 January 2015, between 19 h and 20 h 30 min, in the Parque Estadual da Serra do Mar, Ubatuba (170 km from the species type locality), northern coast of the state of São Paulo, Brazil (23°21′35.34″S 44°50′25.05″W, 10 m a.s.l.). We recorded six unvouchered males using a Tascam recorder model DR-680 with a Sennheiser ME67 directional microphone. All recordings were obtained at 16 bits and 44.1 kHz with the microphone placed at approximately 1 m from the calling individuals. Release calls were elicited by handling one male (pressing the axillary region with fingers) after recording its advertisement call. Animals were captured under a SISBio permit (#42817-2). Our sample size was modest due to the difficulty we had to find many individuals of this species vocalizing in breeding habitats inspected (we recorded all males found). All recordings were deposited in the Fonoteca Neotropical Jacques Vielliard (access codes: FNJV 32363-8). We analyzed the recordings using the software Raven Pro 1.4 (Bioacoustics Research Program, 2011—Cornell Lab of Ornithology). We applied a bandpass filter (lower limit of 500 Hz and upper limit of 4.5 kHz) to decrease background noise. Before the acoustic measurements, calls selected were individually normalized (peak −1.0 dB) using the software Audacity 2.1.1, in order to avoid biases related to variation on call unit intensity. For vocalization analysis, we used the note-centered approach, with calls formed by notes as subunits of the call (Köhler et al., 2017). Temporal properties, such as call duration, note duration and interval between notes were measured in the oscillogram. We measured the following acoustic properties: (1) minimum frequency (Hz), (2) maximum frequency (Hz), (3) peak dominant frequency (Hz), (4) fundamental frequency (Hz) and (5) intensity modulation (dB) using a Fast Fourier Transformation (FFT) of 1024 points and 50% of grid overlap for resolution. We obtained these measurements using the following functions of the “choose measurements” menu: (1) Frequency 5% (Hz); (2) Frequency 95% (Hz); (3) Peak frequency (Hz); and (4) Peak power (dB). For intensity modulation measurements we obtained the peak power of the first and the last note for each call. We generated power spectrum images using software Goldwave v.6.19. For each quantitative acoustic property measured we calculated the average and standard deviation. The variation among and within individuals for each acoustic property was calculated through the coefficient of variation (CV), which is obtained by the following equation: “Standard Deviation/Mean × 100”. We followed the classification of static and dynamic call properties proposed by Gerhardt (1991).

## Results

### *Bioacoustic coldspots* and acoustic data of Lophyohylinae

The subfamily Lophyohylinae includes 39 (out of 85) species with advertisement calls already described, thus 46 (54.1%) species have their vocalizations still undescribed (Table S1). Most of these species occur in the Amazon basin, mainly in the north and western portions, comprising the Guianas, Peru, and Ecuador (Fig. 1A). At the same time these countries comprise hotspot regions for species diversity in the subfamily Lophyohylinae (Fig. 1B). Many of these species belongs to the genus *Osteocephalus* (18 of them).

We found a strong variation in general structure of advertisement calls in the species of the subfamily. Most species (92%) present simple calls composed by one type of note, which in some species (18) are repeated sequentially. Such notes could have a pulsed (64%) or a tonal (36%) structure. Details of quantitative acoustic properties are in Table 1.

### Advertisement call of *Itapotihyla langsdorffii*

**Figure 2:**
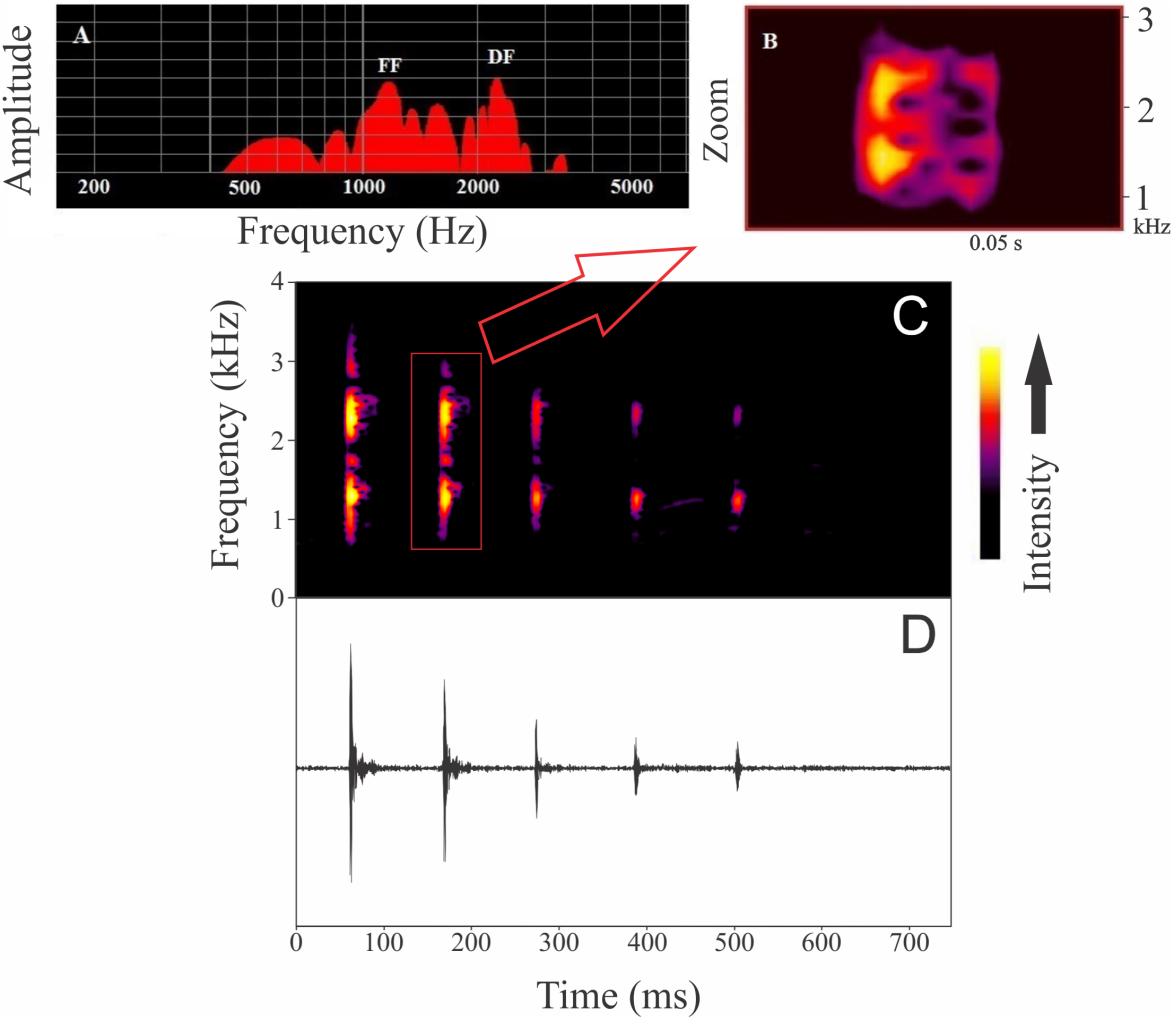
Advertisement call of *Itapotihyla langsdorffii* from Ubatuba. Power spectrum (A) and spectrogram (B) of a single note, and spectrogram (C) and waveform (D) of an advertisement call composed by a sequence of notes of *Itapotihyla langsdorffii* from Ubatuba, Brazil, FNJV 32365. Spectrogram configuration with FFT size = 4,096 samples and 75% window overlap.

**Figure 3:**
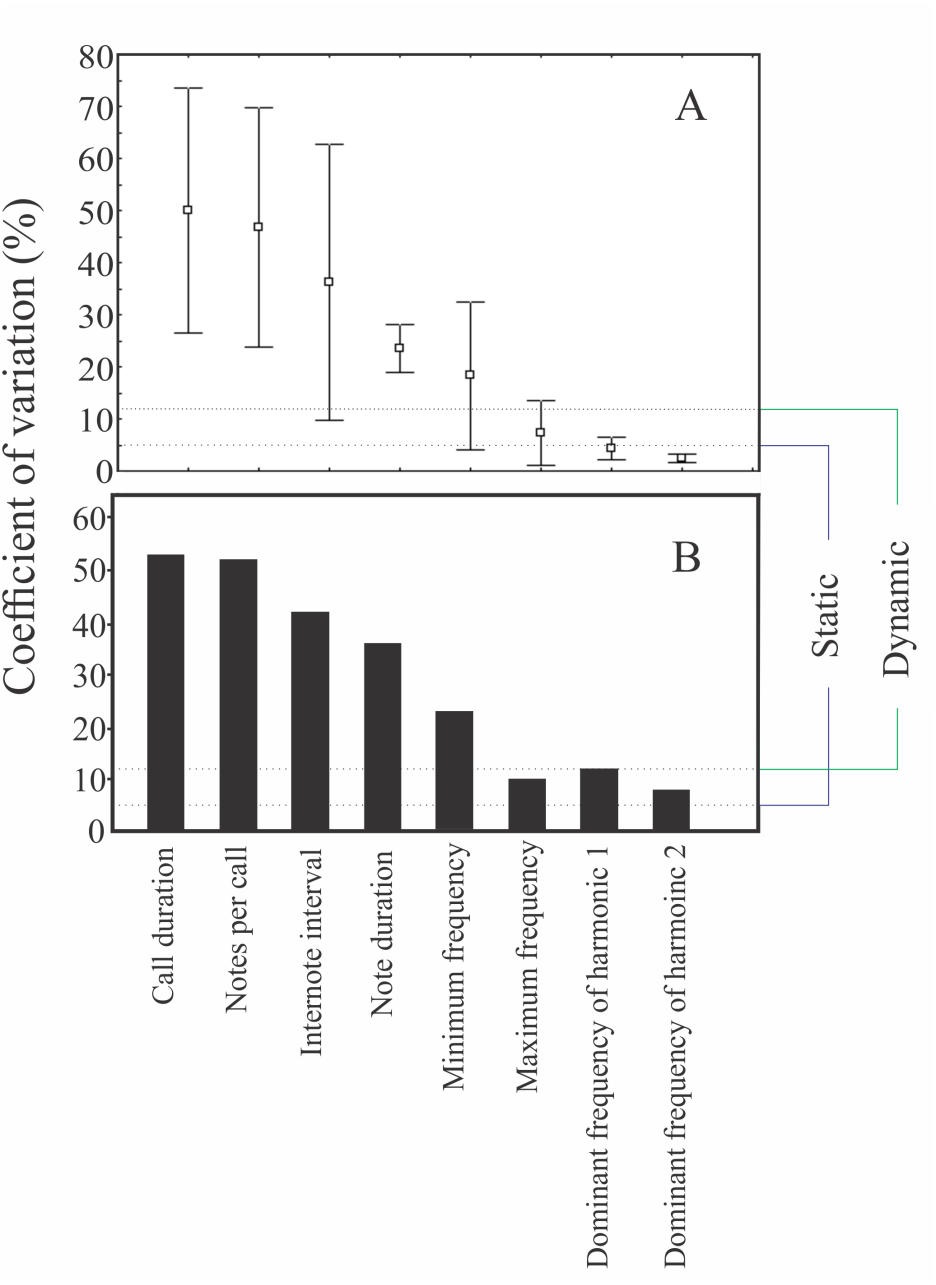
Coefficients of variation of some properties of the advertisement call of *Itapotihyla langsdorffii*. Coefficients of variation of some properties of the advertisement call of *Itapotihyla langsdorffii* at two variation levels: intra-individual (A) and inter-individual (B). The dotted lines represent the 5% and 12% limits to classify static and dynamic properties for intra-individual variation according to Gerhardt (1991).

Males were found calling on leaves of emergent plants and branches of adjacent trees at 101 ± 28 cm from the soil/water surface (*n* = 5). The advertisement call of *Itapotihyla langsdorffii* consists of a sequence of 3–18 notes (*n* = 32) with harmonic structure (Fig. 2). Peak dominant frequency of notes can be in the first or the second harmonic. Most notes (81%) presented peak dominant frequency in the first harmonic (fundamental frequency). The peak dominant frequency of the first harmonic was 1,398 ± 172 Hz (*n* = 149), while it was 2,369 ± 188 Hz (*n* = 35) in the second harmonic. Each note has an average duration of 13 ± 5 ms (ranging from 5 to 31 ms; *n* = 184). In general, notes decrease in intensity (negative intensity modulation) along the call. The average difference in intensity between the first and the last note was 7.8 ± 6 dB (ranging from −5.6 to 17.1 dB; *n* = 31). Average call duration was 615 ± 330 ms (ranging from 265 to 1,939 ms; *n* = 32 calls). The average inter-note interval was 90 ± 40 ms (ranging from 9 to 394 ms; *n* = 144). All temporal properties were considered dynamic (variation above 12%) at both intra- and inter-individual levels. Among spectral properties only peak dominant frequency was recovered as static at intra-individual level. However, peak dominant frequency of the first harmonic presented high variability (12%) at inter-individual level (Fig. 3).

### Male release call of *Itapotihyla langsdorffii*

**Figure 4:**
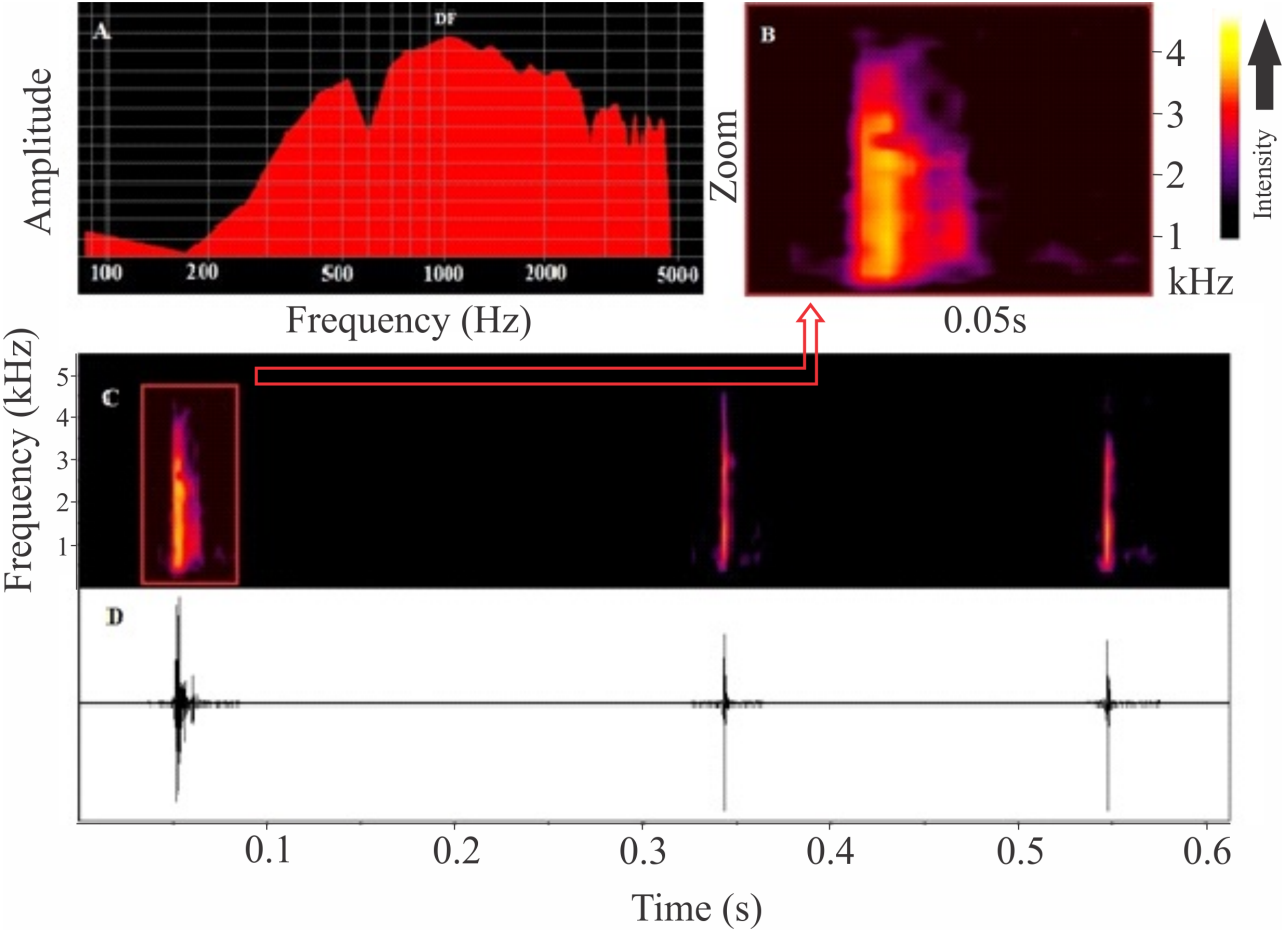
Release calls of *Itapotihyla langsdorffii* from Ubatuba. Power spectrum (A) and spectrogram (B) of a release call, and spectrogram (C) and waveform (D) of a sequence of release calls of *Itapotihyla langsdorffii* from Ubatuba, Brazil, FNJV 3236. Spectrogram configuration with FFT size = 256 samples and 50% window overlap.

The release call of *Itapotihyla langsdorffii* (Fig. 4) is composed of a single and short-unpulsed note with an average duration of 9 ± 2 ms (ranging from 5 to 14 ms; *n* = 52 calls). Notes are click-like. The minimum and maximum frequencies averaged 1,039 ± 187 Hz (ranging from 560 to 1,378 Hz; *n* = 52 calls) and 2,850 ± 281 Hz (ranging from 2,110 to 3,402 Hz; *n* = 52 calls), respectively, and the peak dominant frequency was 1,835 ± 743 Hz (ranging from 1,034 to 3,144; *n* = 52).

## Discussion

Straneck, Olmedo & De Carrizo (1993) briefly reported on the vocalization of *I. langsdorffii* using a recording from Argentina and provided a spectrum figure, but there is no numerical data on spectral and temporal parameters of the call. It is possible to recognize resemblance with the call we described here, as the sequence of harmonic notes (Straneck, Olmedo & De Carrizo, 1993). However, here we present a detailed description of this call, and provide voucher recordings deposited in a scientific sound collection, which enable comparison with other species. The advertisement calls of species in the subfamily Lophyohylinae may vary from simple (one note per call) to complex calls (more than one note per call). Among the species with multi-note advertisement calls, similar intensity modulation to that of *I. langsdorffii* was found in *Osteocephalus leprieurii* and *Osteopilus pulchrilineatus* (Jungfer & Hödl, 2002; Galvis et al., 2016).

Bioacoustic data are helpful in integrative taxonomy (Padial et al., 2010; Köhler et al., 2017), often allowing a better resolution of species delimitations. Extensive bioacoustic comparisons in monophyletic groups may help to establish acoustic boundaries among species and a better understanding of how acoustic signals evolved. However, in the case of the subfamily Lophyohylinae, this endeavor is drastically limited by the low number of species for which advertisement calls are reported.

Most species of the genera *Aparasphenodon* (80%) and *Osteocephalus* (75%) have undescribed calls, while species from other genera, such as *Phyllodytes* and *Tepuihyla* have a lower percentage (19 and 44% respectively) of species with undescribed advertisement calls (see Table 1). Some species may be more easily accessible (as those in the Atlantic forest), when compared to those in the middle of Amazonia. In order to reduce such gaps in the Amazon, future efforts should focus on species from Peru, Ecuador and the Guianas. The genus *Osteocephalus*, for example, has a large number of underestimated species (Jungfer et al., 2013) and new call descriptions will help to better understand the real diversity of this genus.

The release call of *I. langsdorffii* is similar to that of *Trachycephalus cunauaru*: in both species this signal presents short unpulsed notes without harmonic structure, but with large range of frequencies (Gordo et al., 2013). The acoustic simplicity of this call appears to be a universal feature of frog release calls (Stănescu et al., 2018), possibly because, unlike for advertisement calls, release calls may face convergent evolution among species (Leary, 2001). However, release calls are unknown for all other species of the subfamily Lophyohylinae, and should be the subject of further research, given that this signal may provide relevant taxonomic and phylogenetic information (Brown & Littlejohn, 1972; Di Tada, Martino & Sinsch, 2001; Köhler et al., 2017).

Temporal and spectral properties of anuran calls have different levels of variation (Gerhardt, 1991; Gerhardt & Huber, 2002). Usually, in anuran species the spectral properties show low intra-individual variation and are considered static (CV < 5%), while temporal properties may have higher variation and are considered dynamic (CV > 12%) (Márquez & Eekhout, 2006; Forti, Strüssman & Mott, 2010; Briggs, 2010; Morais et al., 2012; Carvalho et al., 2013; Forti, Márquez & Bertoluci, 2015; Forti et al., 2016; Köhler et al., 2017). The variation in acoustic properties of *I. langsdorffii* calls corroborates this pattern, with spectral variables presenting lower variation than temporal ones. This pattern is related to the fact that spectral properties are generally used for species recognition, and the auditory frequency sensibility often matches the frequency range of the conspecific advertisement call (Capranica, Frishkoff & Nevo, 1973). Temporal components of the advertisement call, on the other hand, may be subjected to changes regarding social environment and temperature (Gerhardt, 1991; Gerhardt & Huber, 2002).

## Conclusion

Here we used the term “bioacoustic coldspots” to designate sites with high diversity of species lacking advertisement call descriptions. Based on our spatial analysis in Lophyohylinae, we highlight that recording efforts should be concentrated in the western and northern Amazon regions. Our work presents comparisons of advertisement calls of the Lophyohylinae species, including the description of the advertisement and release calls of *I. langdorffii*, a still poorly studied anuran with a wide distribution. This taxonomic group presents a diversified call structure, varying from simple calls with one or few notes to complex calls presenting a sequence of different notes. Our study may guide future studies targeting those species without proper advertisement call descriptions.

##  Supplemental Information

10.7717/peerj.4813/supp-1 Data S1 Traits of advertisement call of *Itapotihyla langsdorffii*

10.7717/peerj.4813/supp-2 Table S1 List of species with undescribed calls in the subfamily Lophyohylinae, and their Biome of predominant occurrence

## Additional Information and Declarations

### Data Availability

The following information was supplied regarding data availability: Forti. (2018). Current knowledge on bioacoustics of the subfamily Lophyohylinae (Hylidae, Anura) and description of the vocalizations of the ocellated treefrog *Itapotihyla langsdorffii*. http://doi.org/10.5281/zenodo.1183802.
