# Supplementary material for: Current knowledge on bioacoustics of the subfamily Lophyohylinae (Hylidae, Anura) and description of Ocellated treefrog Itapotihyla langsdorffii vocalizations
Source: PeerJ. 2018 May 31;6:e4813. doi: 10.7717/peerj.4813 (PMC5985149; doi:10.7717/peerj.4813)
Supplement: Data S1 [file peerj-06-4813-s001.docx]

Raw data: general traits of advertisement call of *Itapotihyla langsdorffii*

| **Male** | **Internote interval (s)** |
| --- | --- |
| 1 | 0,119 |
| 1 | 0,091 |
| 1 | 0,082 |
| 1 | 0,084 |
| 1 | 0,071 |
| 1 | 0,071 |
| 1 | 0,08 |
| 1 | 0,08 |
| 1 | 0,063 |
| 1 | 0,084 |
| 1 | 0,076 |
| 1 | 0,078 |
| 1 | 0,071 |
| 1 | 0,065 |
| 1 | 0,073 |
| 1 | 0,067 |
| 1 | 0,067 |
| 1 | 0,089 |
| 1 | 0,067 |
| 1 | 0,084 |
| 1 | 0,067 |
| 1 | 0,075 |
| 1 | 0,069 |
| 1 | 0,075 |
| 1 | 0,084 |
| 1 | 0,082 |
| 1 | 0,052 |
| 2 | 0,1 |
| 2 | 0,101 |
| 2 | 0,057 |
| 2 | 0,035 |
| 2 | 0,058 |
| 2 | 0,029 |
| 2 | 0,065 |
| 2 | 0,032 |
| 2 | 0,066 |
| 2 | 0,07 |
| 2 | 0,207 |
| 2 | 0,106 |
| 2 | 0,107 |
| 2 | 0,101 |
| 2 | 0,094 |
| 2 | 0,102 |
| 2 | 0,087 |
| 2 | 0,173 |
| 2 | 0,075 |
| 2 | 0,09 |
| 2 | 0,079 |
| 2 | 0,074 |
| 2 | 0,098 |
| 2 | 0,093 |
| 2 | 0,093 |
| 2 | 0,09 |
| 2 | 0,09 |
| 2 | 0,108 |
| 2 | 0,147 |
| 2 | 0,099 |
| 2 | 0,091 |
| 2 | 0,112 |
| 2 | 0,099 |
| 2 | 0,098 |
| 2 | 0,11 |
| 2 | 0,035 |
| 2 | 0,059 |
| 2 | 0,026 |
| 2 | 0,109 |
| 2 | 0,126 |
| 2 | 0,102 |
| 2 | 0,097 |
| 2 | 0,091 |
| 2 | 0,09 |
| 2 | 0,109 |
| 2 | 0,094 |
| 2 | 0,099 |
| 2 | 0,113 |
| 2 | 0,111 |
| 2 | 0,202 |
| 2 | 0,095 |
| 2 | 0,1 |
| 2 | 0,104 |
| 2 | 0,095 |
| 2 | 0,106 |
| 2 | 0,131 |
| 2 | 0,113 |
| 2 | 0,097 |
| 2 | 0,116 |
| 2 | 0,096 |
| 2 | 0,088 |
| 2 | 0,095 |
| 2 | 0,067 |
| 2 | 0,037 |
| 2 | 0,046 |
| 2 | 0,099 |
| 2 | 0,093 |
| 2 | 0,127 |
| 3 | 0,11 |
| 3 | 0,094 |
| 3 | 0,106 |
| 3 | 0,106 |
| 3 | 0,112 |
| 3 | 0,114 |
| 3 | 0,106 |
| 3 | 0,1 |
| 3 | 0,105 |
| 3 | 0,098 |
| 3 | 0,107 |
| 3 | 0,093 |
| 3 | 0,092 |
| 3 | 0,106 |
| 3 | 0,103 |
| 3 | 0,108 |
| 3 | 0,086 |
| 3 | 0,084 |
| 3 | 0,096 |
| 3 | 0,097 |
| 3 | 0,394 |
| 3 | 0,095 |
| 3 | 0,092 |
| 3 | 0,225 |
| 3 | 0,096 |
| 3 | 0,106 |
| 3 | 0,095 |
| 3 | 0,107 |
| 3 | 0,139 |
| 4 | 0,089 |
| 4 | 0,086 |
| 4 | 0,082 |
| 4 | 0,026 |
| 4 | 0,009 |
| 4 | 0,028 |
| 4 | 0,061 |
| 4 | 0,011 |
| 4 | 0,133 |
| 5 | 0,097 |
| 5 | 0,095 |
| 5 | 0,092 |
| 5 | 0,076 |
| 5 | 0,088 |
| 5 | 0,087 |
| 5 | 0,088 |
| 5 | 0,095 |
| 5 | 0,094 |
| 5 | 0,098 |
| 5 | 0,097 |

Raw data: note traits of advertisement call of *Itapotihyla langsdorffii*

| **Male** | **Call** | **Note duration (s)** | **Mininum Frequency (Hz)** | **Maximum Frequency (Hz)** | **Dominant Frequency (Hz) harm 1** | **Harmonic dominant** | **Dominant Frequency (Hz) Harm 2** |
| --- | --- | --- | --- | --- | --- | --- | --- |
| 1 | 1 | 0,019 | 1031,2 | 2625 | 1359,4 | 1 |  |
| 1 | 1 | 0,013 | 1078,1 | 2531,2 | 1312,5 | 1 |  |
| 1 | 1 | 0,014 | 1125 | 2578,1 | 1265,6 | 1 |  |
| 1 | 1 | 0,019 | 984,4 | 2437,5 | 1312,5 | 1 |  |
| 1 | 2 | 0,021 | 1078,1 | 2578,1 | 1312,5 | 1 |  |
| 1 | 2 | 0,018 | 1171,9 | 2531,2 | 1312,5 | 1 |  |
| 1 | 2 | 0,016 | 984,4 | 2531,2 | 1312,5 | 1 |  |
| 1 | 2 | 0,018 | 609,4 | 2671,9 | 1406,2 | 1 |  |
| 1 | 2 | 0,027 | 234,4 | 2812,5 | 1359,4 | 1 |  |
| 1 | 2 | 0,026 | 93,8 | 2625 | 1359,4 | 1 |  |
| 1 | 2 | 0,021 | 140,6 | 2484,4 | 1406,2 | 1 |  |
| 1 | 2 | 0,023 | 937,5 | 2718,8 | 1406,2 | 1 |  |
| 1 | 3 | 0,021 | 796,9 | 2296,9 | 1500 | 1 |  |
| 1 | 3 | 0,026 | 984,4 | 2296,9 | 1453,1 | 1 |  |
| 1 | 3 | 0,027 | 46,9 | 2437,5 | 1406,2 | 1 |  |
| 2 | 1 | 0,012 | 1248,9 | 2540,9 | 1378,1 | 1 |  |
| 2 | 1 | 0,011 | 947,5 | 2627,1 | 1464,3 | 1 |  |
| 2 | 1 | 0,008 | 1205,9 | 2368,7 | 1335,1 | 1 |  |
| 2 | 2 | 0,01 | 1248,9 | 2713,2 | 1464,3 | 1 |  |
| 2 | 2 | 0,007 | 1421,2 | 2411,7 | 1593,5 | 1 |  |
| 2 | 2 | 0,005 | 1248,9 | 2239,5 | 1593,5 | 1 |  |
| 2 | 2 | 0,008 | 1119,7 | 2454,8 | 1464,3 | 1 |  |
| 2 | 2 | 0,007 | 1162,8 | 2368,7 | 1378,1 | 1 |  |
| 2 | 2 | 0,008 | 904,4 | 2670,1 | 1378,1 | 1 |  |
| 2 | 3 | 0,01 | 904,4 | 2670,1 | 1507,3 | 1 |  |
| 2 | 3 | 0,017 | 1292 | 2411,7 | 1550,4 | 1 |  |
| 2 | 3 | 0,01 | 1421,2 | 2411,7 | 1593,5 | 1 |  |
| 2 | 4 | 0,012 | 1378,1 | 2239,5 | 1593,5 | 1 |  |
| 2 | 4 | 0,012 | 1205,9 | 2497,9 | 1335,1 | 1 |  |
| 2 | 4 | 0,011 | 904,4 | 2928,5 | 1679,6 | 1 |  |
| 2 | 4 | 0,009 | 861,3 | 3014,6 | 1679,6 | 1 |  |
| 2 | 5 | 0,015 | 689,1 | 3014,6 | 1593,5 | 1 |  |
| 2 | 5 | 0,015 | 1248,9 | 2928,5 | 1636,5 | 1 |  |
| 2 | 5 | 0,015 | 1162,8 | 2971,6 | 1636,5 | 1 |  |
| 2 | 5 | 0,023 | 689,1 | 2885,4 | 1507,3 | 1 |  |
| 2 | 5 | 0,018 | 1421,2 | 2799,3 | 1593,5 | 1 |  |
| 2 | 6 | 0,018 | 1248,9 | 2670,1 | 1421,2 | 1 |  |
| 2 | 6 | 0,014 | 1076,7 | 2756,2 | 1378,1 | 1 |  |
| 2 | 7 | 0,012 | 1205,9 | 2670,1 | 1421,2 | 1 |  |
| 2 | 7 | 0,01 | 1162,8 | 2411,7 | 1335,1 | 1 |  |
| 2 | 7 | 0,01 | 1205,9 | 2411,7 | 1335,1 | 1 |  |
| 2 | 7 | 0,009 | 1464,3 | 2627,1 | 1593,5 | 1 |  |
| 2 | 7 | 0,006 | 1421,2 | 2670,1 | 1593,5 | 1 |  |
| 2 | 8 | 0,014 | 1378,1 | 2584 | 1593,5 | 1 |  |
| 2 | 8 | 0,014 | 1378,1 | 2627,1 | 1593,5 | 1 |  |
| 2 | 8 | 0,013 | 1292 | 2196,4 | 1593,5 | 1 |  |
| 2 | 9 | 0,014 | 990,5 | 2540,9 | 1464,3 | 1 |  |
| 2 | 9 | 0,015 | 1248,9 | 2584 | 1593,5 | 1 |  |
| 2 | 9 | 0,009 | 1033,6 | 2627,1 | 1507,3 | 1 |  |
| 2 | 10 | 0,008 | 1378,1 | 2239,5 | 1593,5 | 1 |  |
| 2 | 10 | 0,01 | 1335,1 | 2239,5 | 1593,5 | 1 |  |
| 2 | 10 | 0,01 | 818,3 | 2239,5 | 1378,1 | 1 |  |
| 2 | 10 | 0,014 | 1248,9 | 2497,9 | 1378,1 | 1 |  |
| 2 | 11 | 0,013 | 1205,9 | 2713,2 | 1464,3 | 1 |  |
| 2 | 11 | 0,013 | 1248,9 | 2454,8 | 1421,2 | 1 |  |
| 2 | 12 | 0,012 | 1464,3 | 2885,4 | 1765,7 | 1 |  |
| 2 | 12 | 0,01 | 1335,1 | 2842,4 | 1808,8 | 1 |  |
| 2 | 12 | 0,008 | 1292 | 2713,2 | 1593,5 | 1 |  |
| 2 | 12 | 0,014 | 1464,3 | 2756,2 | 1507,3 | 1 |  |
| 2 | 13 | 0,009 | 947,5 | 2756,2 | 1593,5 | 1 |  |
| 2 | 13 | 0,006 | 1162,8 | 2713,2 | 1550,4 | 1 |  |
| 2 | 13 | 0,007 | 689,1 | 2670,1 | 1507,3 | 1 |  |
| 2 | 14 | 0,01 | 1033,6 | 2713,2 | 1507,3 | 1 |  |
| 2 | 14 | 0,009 | 1292 | 2540,9 | 1335,1 | 1 |  |
| 2 | 15 | 0,009 | 904,4 | 2584 | 1421,2 | 1 |  |
| 2 | 15 | 0,007 | 1119,7 | 2540,9 | 1378,1 | 1 |  |
| 2 | 15 | 0,006 | 1076,7 | 2497,9 | 1335,1 | 1 |  |
| 2 | 15 | 0,012 | 775,2 | 2497,9 | 1335,1 | 1 |  |
| 2 | 15 | 0,009 | 602,9 | 2497,9 | 1335,1 | 1 |  |
| 2 | 15 | 0,012 | 1033,6 | 2670,1 | 1550,4 | 1 |  |
| 2 | 15 | 0,008 | 1378,1 | 2584 | 1507,3 | 1 |  |
| 2 | 16 | 0,008 | 1119,7 | 2627,1 | 1507,3 | 1 |  |
| 2 | 16 | 0,016 | 646 | 2540,9 | 1378,1 | 1 |  |
| 2 | 16 | 0,01 | 1248,9 | 2368,7 | 1464,3 | 1 |  |
| 2 | 16 | 0,01 | 1248,9 | 2540,9 | 1335,1 | 1 |  |
| 2 | 17 | 0,017 | 1248,9 | 2454,8 | 1593,5 | 1 |  |
| 2 | 17 | 0,01 | 1205,9 | 2627,1 | 1335,1 | 1 |  |
| 2 | 17 | 0,02 | 1335,1 | 2153,3 | 1593,5 | 1 |  |
| 2 | 17 | 0,009 | 1076,7 | 2411,7 | 1593,5 | 1 |  |
| 2 | 17 | 0,016 | 1248,9 | 2627,1 | 1464,3 | 1 |  |
| 2 | 17 | 0,015 | 602,9 | 2325,6 | 1679,6 | 1 |  |
| 2 | 17 | 0,008 | 1119,7 | 2282,5 | 1421,2 | 1 |  |
| 2 | 17 | 0,009 | 689,1 | 1981,1 | 1679,6 | 1 |  |
| 2 | 17 | 0,012 | 689,1 | 2196,4 | 1636,5 | 1 |  |
| 2 | 17 | 0,01 | 689,1 | 2497,9 | 1464,3 | 1 |  |
| 2 | 18 | 0,008 | 1205,9 | 2368,7 | 1335,1 | 1 |  |
| 2 | 18 | 0,01 | 1119,7 | 2713,2 | 1335,1 | 1 |  |
| 2 | 18 | 0,009 | 646 | 2411,7 | 1335,1 | 1 |  |
| 2 | 18 | 0,008 | 689,1 | 2024,1 | 1335,1 | 1 |  |
| 2 | 19 | 0,016 | 1335,1 | 2670,1 | 1679,6 | 1 |  |
| 2 | 19 | 0,01 | 1421,2 | 2670,1 | 1593,5 | 1 |  |
| 2 | 19 | 0,008 | 990,5 | 2670,1 | 1550,4 | 1 |  |
| 2 | 19 | 0,008 | 990,5 | 2627,1 | 1464,3 | 1 |  |
| 2 | 19 | 0,008 | 1378,1 | 2799,3 | 1636,5 | 1 |  |
| 2 | 19 | 0,013 | 1421,2 | 2540,9 | 1636,5 | 1 |  |
| 2 | 19 | 0,012 | 1076,7 | 2670,1 | 1636,5 | 1 |  |
| 2 | 19 | 0,011 | 1205,9 | 2627,1 | 1464,3 | 1 |  |
| 2 | 19 | 0,01 | 947,5 | 2584 | 1378,1 | 1 |  |
| 2 | 19 | 0,015 | 1248,9 | 2584 | 1335,1 | 1 |  |
| 2 | 20 | 0,012 | 1292 | 2627,1 | 1593,5 | 1 |  |
| 2 | 20 | 0,014 | 1421,2 | 2368,7 | 1593,5 | 1 |  |
| 2 | 20 | 0,012 | 1421,2 | 2368,7 | 1593,5 | 1 |  |
| 2 | 20 | 0,015 | 990,5 | 2928,5 | 1378,1 | 1 |  |
| 3 | 1 | 0,013 | 990,5 | 2239,5 | 1162,8 | 1 |  |
| 3 | 1 | 0,011 | 990,5 | 2239,5 | 1119,7 | 1 |  |
| 3 | 1 | 0,016 | 990,5 | 2239,5 | 1162,8 | 1 |  |
| 3 | 1 | 0,014 | 947,5 | 2196,4 | 1119,7 | 1 |  |
| 3 | 2 | 0,011 | 1119,7 | 2282,5 | 1162,8 | 1 |  |
| 3 | 2 | 0,012 | 1033,6 | 2239,5 | 1162,8 | 1 |  |
| 3 | 2 | 0,013 | 990,5 | 2282,5 | 1119,7 | 1 |  |
| 3 | 2 | 0,013 | 990,5 | 2239,5 | 1162,8 | 1 |  |
| 3 | 2 | 0,013 | 1033,6 | 2239,5 | 1162,8 | 1 |  |
| 3 | 2 | 0,016 | 947,5 | 2153,3 | 1076,7 | 1 |  |
| 3 | 3 | 0,01 | 1033,6 | 2325,6 | 1119,7 | 1 |  |
| 3 | 3 | 0,013 | 990,5 | 2239,5 | 1162,8 | 1 |  |
| 3 | 3 | 0,019 | 689,1 | 2153,3 | 1119,7 | 1 |  |
| 3 | 3 | 0,015 | 904,4 | 2196,4 | 1119,7 | 1 |  |
| 3 | 4 | 0,024 | 947,5 | 2282,5 | 1162,8 | 1 |  |
| 3 | 4 | 0,021 | 990,5 | 2239,5 | 1162,8 | 1 |  |
| 3 | 4 | 0,02 | 947,5 | 2239,5 | 1162,8 | 1 |  |
| 3 | 4 | 0,013 | 904,4 | 2196,4 | 1119,7 | 1 |  |
| 3 | 5 | 0,01 | 1119,7 | 2325,6 | 1162,8 | 1 |  |
| 3 | 5 | 0,014 | 1076,7 | 2282,5 | 1162,8 | 1 |  |
| 3 | 5 | 0,012 | 1033,6 | 2325,6 | 1162,8 | 1 |  |
| 3 | 5 | 0,017 | 990,5 | 2153,3 | 1119,7 | 1 |  |
| 3 | 6 | 0,011 | 1033,6 | 2282,5 | 1162,8 | 1 |  |
| 3 | 6 | 0,014 | 990,5 | 2282,5 | 1119,7 | 1 |  |
| 3 | 6 | 0,01 | 990,5 | 2282,5 | 1162,8 | 1 |  |
| 3 | 6 | 0,014 | 861,3 | 2110,3 | 1162,8 | 1 |  |
| 3 | 6 | 0,015 | 861,3 | 2196,4 | 1033,6 | 1 |  |
| 4 | 1 | 0,012 | 818,3 | 2971,6 | 1378,1 | 1 |  |
| 4 | 1 | 0,01 | 1033,6 | 2799,3 | 1378,1 | 1 |  |
| 4 | 1 | 0,013 | 1033,6 | 2756,2 | 1335,1 | 1 |  |
| 4 | 1 | 0,013 | 1033,6 | 2842,4 | 1378,1 | 1 |  |
| 4 | 1 | 0,013 | 1378,1 | 4048,2 | 1464,3 | 1 |  |
| 4 | 1 | 0,014 | 1076,7 | 3617,6 | 1335,1 | 1 |  |
| 4 | 1 | 0,015 | 1033,6 | 2497,9 | 1378,1 | 1 |  |
| 5 | 1 | 0,014 | 1119,7 | 2325,6 | 1248,9 | 1 |  |
| 5 | 1 | 0,011 | 1076,7 | 2454,8 | 1292 | 1 |  |
| 5 | 1 | 0,011 | 1119,7 | 2282,5 | 1292 | 1 |  |
| 5 | 1 | 0,014 | 1033,6 | 2282,5 | 1248,9 | 1 |  |
| 5 | 1 | 0,014 | 1076,7 | 2239,5 | 1248,9 | 1 |  |
| 5 | 1 | 0,014 | 1076,7 | 2239,5 | 1248,9 | 1 |  |
| 5 | 1 | 0,012 | 1119,7 | 2239,5 | 1248,9 | 1 |  |
| 5 | 1 | 0,013 | 1119,7 | 2239,5 | 1248,9 | 1 |  |
| 5 | 1 | 0,016 | 990,5 | 2239,5 | 1248,9 | 1 |  |
| 5 | 2 | 0,012 | 1205,9 | 2454,8 | 1335,1 | 1 |  |
| 5 | 2 | 0,011 | 1119,7 | 2325,6 | 1292 | 1 |  |
| 5 | 2 | 0,008 | 1033,6 | 2282,5 | 1205,9 | 1 |  |
| 1 | 1 | 0,026 | 1031,2 | 2531,2 |  | 2 | 2343,8 |
| 1 | 1 | 0,023 | 1031,2 | 2578,1 |  | 2 | 2390,6 |
| 1 | 1 | 0,015 | 1171,9 | 2531,2 |  | 2 | 2390,6 |
| 1 | 1 | 0,011 | 1078,1 | 2437,5 |  | 2 | 2390,6 |
| 1 | 2 | 0,031 | 1125 | 2531,2 |  | 2 | 2343,8 |
| 1 | 2 | 0,021 | 1171,9 | 2578,1 |  | 2 | 2390,6 |
| 1 | 2 | 0,027 | 703,1 | 2578,1 |  | 2 | 2390,6 |
| 1 | 2 | 0,016 | 1125 | 2531,2 |  | 2 | 2390,6 |
| 1 | 2 | 0,019 | 1171,9 | 2484,4 |  | 2 | 2390,6 |
| 1 | 2 | 0,016 | 1171,9 | 2484,4 |  | 2 | 2390,6 |
| 1 | 2 | 0,016 | 1171,9 | 2531,2 |  | 2 | 2390,6 |
| 1 | 2 | 0,023 | 656,2 | 2531,2 |  | 2 | 2390,6 |
| 1 | 2 | 0,027 | 468,8 | 2531,2 |  | 2 | 2296,9 |
| 1 | 2 | 0,016 | 1171,9 | 2625 |  | 2 | 2437,5 |
| 2 | 2 | 0,008 | 947,5 | 2627,1 |  | 2 | 2497,9 |
| 2 | 5 | 0,012 | 1248,9 | 2971,6 |  | 2 | 2627,1 |
| 2 | 6 | 0,014 | 904,4 | 2799,3 |  | 2 | 2627,1 |
| 2 | 7 | 0,009 | 947,5 | 2713,2 |  | 2 | 2497,9 |
| 2 | 8 | 0,014 | 1205,9 | 2584 |  | 2 | 2497,9 |
| 2 | 9 | 0,008 | 1119,7 | 2713,2 |  | 2 | 2497,9 |
| 2 | 11 | 0,012 | 1076,7 | 3014,6 |  | 2 | 2799,3 |
| 2 | 13 | 0,01 | 1248,9 | 2799,3 |  | 2 | 2584 |
| 2 | 14 | 0,008 | 646 | 2885,4 |  | 2 | 2756,2 |
| 3 | 1 | 0,018 | 947,5 | 2239,5 |  | 2 | 2110,3 |
| 3 | 1 | 0,012 | 1033,6 | 2239,5 |  | 2 | 2110,3 |
| 3 | 1 | 0,014 | 1033,6 | 2196,4 |  | 2 | 2110,3 |
| 3 | 3 | 0,02 | 904,4 | 2325,6 |  | 2 | 2067,2 |
| 3 | 3 | 0,018 | 1033,6 | 2411,7 |  | 2 | 2067,2 |
| 3 | 4 | 0,015 | 1076,7 | 2282,5 |  | 2 | 2196,4 |
| 3 | 4 | 0,012 | 1033,6 | 2411,7 |  | 2 | 2067,2 |
| 3 | 6 | 0,014 | 947,5 | 2411,7 |  | 2 | 2110,3 |
| 4 | 1 | 0,017 | 1335,1 | 3660,6 |  | 2 | 2282,5 |
| 4 | 1 | 0,015 | 1378,1 | 2627,1 |  | 2 | 2411,7 |
| 4 | 1 | 0,007 | 1205,9 | 2540,9 |  | 2 | 2411,7 |
| 5 | 2 | 0,01 | 1119,7 | 2411,7 |  | 2 | 2239,5 |

Raw data: traits of release call of *Itapotihyla langsdorffii*

| **Male** | **Duration call (s)** | **Internote interval (s)** | **Mininum Frequency (Hz)** | **Maximum Frequency (Hz)** | **Dominant Frequency (Hz)** | **Call rate (calls/min)** |
| --- | --- | --- | --- | --- | --- | --- |
| 1 | 0,005 | 0,20 | 1249 | 3402 | 3144 | 26 |
| 1 | 0,008 | 0,15 | 1163 | 3273 | 3144 | 26 |
| 1 | 0,008 | 0,32 | 1163 | 3058 | 1335 | 26 |
| 1 | 0,011 | 0,14 | 1163 | 3058 | 2929 | 26 |
| 1 | 0,009 | 0,15 | 1206 | 3101 | 1421 | 26 |
| 1 | 0,006 | 0,26 | 1120 | 3015 | 1292 | 26 |
| 1 | 0,007 | 0,01 | 948 | 2196 | 1292 | 26 |
| 1 | 0,010 | 0,79 | 1335 | 3187 | 3058 | 26 |
| 1 | 0,008 | 0,12 | 1163 | 3101 | 2972 | 26 |
| 1 | 0,013 | 0,59 | 603 | 2756 | 1249 | 26 |
| 1 | 0,010 | 0,17 | 1206 | 2799 | 1378 | 26 |
| 1 | 0,008 | 0,49 | 1206 | 2412 | 1378 | 26 |
| 1 | 0,010 | 0,03 | 991 | 2110 | 1464 | 26 |
| 1 | 0,009 | 0,25 | 1292 | 2842 | 1464 | 26 |
| 1 | 0,008 | 0,64 | 1077 | 2498 | 1464 | 26 |
| 1 | 0,009 | 0,36 | 1163 | 2756 | 2498 | 26 |
| 1 | 0,008 | 0,21 | 1292 | 2885 | 2670 | 26 |
| 1 | 0,009 | 0,93 | 1249 | 2972 | 2713 | 26 |
| 1 | 0,008 | 1,28 | 1378 | 2842 | 2713 | 26 |
| 1 | 0,008 | 0,63 | 1034 | 2240 | 1550 | 26 |
| 1 | 0,009 | 3,05 | 1292 | 2842 | 2584 | 26 |
| 1 | 0,007 | 0,14 | 1120 | 2713 | 2455 | 26 |
| 1 | 0,009 | 0,26 | 1034 | 2670 | 1852 | 26 |
| 1 | 0,008 | 0,67 | 1034 | 2498 | 1723 | 26 |
| 1 | 0,007 | 64,36 | 1335 | 2670 | 2412 | 26 |
| 1 | 0,013 | 0,22 | 560 | 2885 | 1120 | 26 |
| 1 | 0,007 | 0,15 | 1034 | 2756 | 1249 | 26 |
| 1 | 0,010 | 0,46 | 646 | 2498 | 1163 | 26 |
| 1 | 0,009 | 0,31 | 1034 | 2584 | 1421 | 26 |
| 1 | 0,009 | 0,39 | 948 | 2929 | 1077 | 26 |
| 1 | 0,008 | 1,79 | 991 | 2369 | 1249 | 26 |
| 1 | 0,008 | 0,39 | 689 | 2842 | 1120 | 26 |
| 1 | 0,014 | 0,44 | 904 | 2756 | 1163 | 26 |
| 1 | 0,010 | 0,76 | 948 | 2929 | 1163 | 26 |
| 1 | 0,009 | 1,74 | 1120 | 3101 | 2713 | 26 |
| 1 | 0,006 | 1,57 | 991 | 2885 | 1163 | 26 |
| 1 | 0,008 | 0,41 | 991 | 2972 | 2842 | 26 |
| 1 | 0,009 | 0,42 | 1077 | 2929 | 2799 | 26 |
| 1 | 0,008 | 0,46 | 1077 | 3015 | 2929 | 26 |
| 1 | 0,007 | 0,50 | 948 | 2756 | 2627 | 26 |
| 1 | 0,008 | 3,25 | 948 | 3101 | 1120 | 26 |
| 1 | 0,010 | 0,27 | 861 | 2756 | 1077 | 26 |
| 1 | 0,006 | 1,30 | 818 | 3101 | 1077 | 26 |
| 1 | 0,008 | 2,57 | 991 | 3101 | 1120 | 26 |
| 1 | 0,008 | 0,37 | 948 | 2799 | 1378 | 26 |
| 1 | 0,008 | 0,34 | 1120 | 3058 | 2885 | 26 |
| 1 | 0,007 | 0,44 | 948 | 3058 | 1034 | 26 |
| 1 | 0,009 | 0,41 | 991 | 2799 | 1378 | 26 |
| 1 | 0,008 | 1,01 | 1077 | 2972 | 2498 | 26 |
| 1 | 0,007 |  | 689 | 3273 | 1507 | 26 |
| 1 | 0,013 |  | 948 | 3316 | 1034 | 26 |
| 1 | 0,009 |  | 904 | 2756 | 1378 | 26 |
