# Supplementary material for: Current knowledge on bioacoustics of the subfamily Lophyohylinae (Hylidae, Anura) and description of Ocellated treefrog Itapotihyla langsdorffii vocalizations
Source: PeerJ. 2018 May 31;6:e4813. doi: 10.7717/peerj.4813 (PMC5985149; doi:10.7717/peerj.4813)
Supplement: Table S1 [file peerj-06-4813-s002.pdf]

Table S1 – List of species with undescribed calls in the subfamily Lophyohylinae, and their Biome of predominant occurrence.

**Species**	**Biome**
Aparasphenodon bokermanni Pombal, 1993	Atlantic forest
Aparasphenodon brunoi Miranda-Ribeiro, 1920	Atlantic forest
Aparasphenodon pomba Assis, Santana, Silva, Quintela, and Feio, 2013	Atlantic forest
Aparasphenodon venezolanus (Mertens, 1950)	Amazon
Corythomantis galeata Pombal, Menezes, Fontes, Nunes, Rocha, and Van Sluys, 2012	Caatinga
Dryaderces inframaculata (Boulenger, 1882)	Amazon
Dryaderces pearsoni (Gaige, 1929)	Amazon
Osteocephalus alboguttatus (Boulenger, 1882)	Amazon
Osteocephalus cabrerai (Cochran and Goin, 1970)	Amazon
Osteocephalus camufatus Jungfer, Verdade, Faivovich, and Rodrigues, 2016	Amazon
Osteocephalus carri (Cochran and Goin, 1970)	Amazon
Osteocephalus castaneicola Moravec, Aparicio, Guerrero-Reinhard, Calderón, Jungfer, and Gvoždík, 2009	Amazon
Osteocephalus duellmani Jungfer, 2011	Amazon
Osteocephalus festae (Peracca, 1904)	Amazon
Osteocephalus fuscifacies Jungfer, Ron, Seipp, and Almendáriz, 2000	Amazon
Osteocephalus helenae (Ruthven, 1919)	Amazon
Osteocephalus heyeri Lynch, 2002	Amazon
Osteocephalus leoniae Jungfer and Lehr, 2001	Amazon
Osteocephalus mimeticus (Melin, 1941)	Amazon
Osteocephalus oophagus Jungfer and Schiesari, 1995	Amazon
Osteocephalus planiceps Cope, 1874	Amazon
Osteocephalus subtilis Martins and Cardoso, 1987	Amazon
Osteocephalus verruciger (Werner, 1901)	Amazon
Osteocephalus vilarsi (Melin, 1941)	Amazon
Osteocephalus yasuni Ron and Pramuk, 1999	Amazon
Osteopilus crucialis (Harlan, 1826)	Caribbean forest
Osteopilus ocellatus (Linnaeus, 1758)	Caribbean forest

Osteopilus vastus (Cope, 1871)	Caribbean forest
Osteopilus wilderi (Dunn, 1925)	Caribbean forest
Phyllodytes brevirostris Peixoto and Cruz, 1988	Atlantic forest
Phyllodytes maculosus Cruz, Feio, and Cardoso, 2007	Atlantic forest
Phyllodytes punctatus Caramaschi and Peixoto, 2004	Atlantic forest
Phytotriades auratus (Boulenger, 1917)	Amazon
Tepuihyla aecii Ayarzagüena, Señaris, and Gorzula, 1993	Amazon
Tepuihyla exophthalma (Smith and Noonan, 2001)	Amazon
Tepuihyla luteolabris Ayarzagüena, Señaris, and Gorzula, 1993	Amazon
Tepuihyla warreni (Duellman and Hoogmoed, 1992)	Amazon
Trachycephalus "vermiculatus" (Cope, 1877)	Caribbean forest
Trachycephalus coriaceus (Peters, 1867)	Amazon
Trachycephalus hadroceps (Duellman and Hoogmoed, 1992)	Amazon
Trachycephalus helioi Nunes, Suárez, Gordo, and Pombal, 2013	Amazon
Trachycephalus jordani (Stejneger and Test, 5891)	Amazon
Trachycephalus lepidus (Pombal, Haddad, and Cruz, 2003)	Atlantic forest
Trachycephalus macrotis (Andersson, 1945)	Amazon
Trachycephalus mambaiensis Cintra, Silva, Silva, Garcia, and Zaher, 2009	savannah
Trachycephalus quadrangulum (Boulenger, 1882)	Amazon
